# Characteristics and in-hospital morbidity trends associated with oral and oropharyngeal cancers in Brazil

**DOI:** 10.1186/s12903-022-02312-0

**Published:** 2022-07-06

**Authors:** Deborah Gomes de Miranda Vargas, Amanda Ramos da Cunha, Livia Fernandes Probst, Edílson José Zafalon, Paulo Zárate Pereira, Elaine Pereira da Silva Tagliaferro, Alessandro Diogo De-Carli

**Affiliations:** 1 Programa de Pós-Graduação em Saúde da Família, Universidade Federal de Mato Grosso do Sul (UFMS), Campo Grande, Brazil; 2 Faculdade de Odontologia, Universidade Federal do Rio Grande do Sul (UFRGS), Porto Alegre, Brazil; 3 Unidade de Avaliação de Tecnologias em Saúde, Hospital Alemão Oswaldo Cruz (HAOC), São Paulo, Brazil; 4 Faculdade de Odontologia, Universidade Federal de Mato Grosso do Sul (UFMS), Campo Grande, Brazil; 5 Faculdade de Odontologia de Araraquara, Universidade Estadual Paulista (Unesp), Araraquara, Brazil

**Keywords:** Oral neoplasms, Oropharyngeal neoplasms, Temporal series studies, Hospital records

## Abstract

**Background:**

Brazil experienced an expansion of the population's access to oral health policies after the creation of the Unified Health System (SUS, Sistema Único de Saúde). Through public policies, the consolidation of Primary Health Care (PHC) and the incorporation of dental care into primary and hospital care took place. The objective of this study was to identify epidemiological aspects, including the temporal trend, of hospital morbidity from oral and oropharyngeal cancer in Brazil, considering hospitalizations for this neoplasm in a hospital network linked to the public care system.

**Methods:**

Observational study based on information on hospital admissions for oral cancer throughout Brazil. The research used data from the Brazilian Cancer Registry Information System. For the temporal series analysis, generalized linear regression model was used with the Prais-Winsten method.

**Results:**

Of the 121,971 patients hospitalized with oral and oropharyngeal cancers, 76.40% were male and 23.60% were female, resulting in a M:F ratio of 3.24:1. Regarding the anatomical region of involvement among hospitalized patients with oral cavity neoplastic lesions, there was a predominance in non-specific places in the mouth, such as the floor of the mouth, soft and hard palate, among others (32.68%), followed by lesions in the region of tongue (28.89%). In this population, the predominant age group was between the fifth decade (31.09%) and sixth decade of life (24.99%); men presented neoplastic lesions of oral and oropharyngeal cancers at an earlier age than women. In all regions of the country, the staging of cases diagnosed in the tertiary health network accredited to the José Alencar Gomes da Silva National Cancer Institute (INCA) was late, with higher tendency for metastasis. The temporal trend of the adjusted in-hospital morbidity rates showed to be increasing in the Northeast, South and Midwest regions for the male gender. For females, they were increasing in the Northeast and South regions.

**Conclusions:**

It is concluded that the distribution of in-hospital morbidity rates of oral and oropharyngeal cancers in the country is irregular. There is a greater number of cases identified by the study in male patients and in the Southeast and South regions; with an increasing tendency of this coefficient in both genders.

## Introduction

Neoplastic lesions primarily located in the lips, oral cavity, salivary glands and oropharynx are classified as oral and oropharyngeal cancers, according to the José de Alencar National Cancer Institute (INCA, Instituto Nacional do Câncer) [1, 2].

In Brazil, there are an estimated 11,180 new cases of oral and oropharyngeal cancer in men and 4100 in women for each year of the 2020–2022 triennium. In males it is the 5th most frequent and in females it is the 13th most frequent cancer among all neoplasms. There is also a growing incidence of this disease in younger people and women [3].

Some risk factors are common for oral and oropharyngeal cancers, such as the habits of smoking/chewing tobacco and drinking alcohol above the recommended levels, especially when they are associated [4, 5]. In addition to these factors, human papillomavirus (HPV) is an important etiological factor for oropharyngeal cancers, being recognized as the main risk factor in some regions of the world [6, 7]. HPV-associated oropharyngeal neoplasms are considered to be clinical entities distinct from those not associated with HPV. It is observed that the etiology, clinical behavior, treatment indications, response to treatment and survival are different from those not associated ones [8, 9]. However, in Brazil, HPV does not yet seem to be the main risk factor for oropharyngeal cancer [10].

In turn, an early diagnosis favors the implementation of usually less invasive and more effective treatments [5]. Behaviors associated to primary prevention, early detection and adequate and timely treatment are associated, among other factors, to the organization and quality of the provided health services [11, 12]. In fact, the incorporation of the monitoring of morbidity and mortality from cancer in the health management routine is of utmost importance to implement actions aimed at preventing and controlling cancer and its risk factors [13, 14].

Brazil experienced an expansion of the population with access to oral health policies after the creation of the Unified Health System (SUS, Sistema Único de Saúde), the consolidation of Primary Health Care (PHC) and the incorporation of dental care in both primary and hospital care [14–16]. Furthermore, the National Cancer Prevention and Control Policy establishes that cancer treatment in Brazil should be carried out in qualified health facilities. In addition, these institutions must transmit information from the data from the hospital records to the Integrator Module of Hospital Cancer Records (RHC Integrator) [17].

Considering the scarcity of information and in order to characterize the variations in the burden of disease by Brazilian macro-region, it was decided to study time series of hospital morbidity from oral and oropharyngeal cancer. The trend analysis for this indicator, in the evaluated period, is unprecedented in Brazil. Therefore, the objective of this study was to analyze epidemiological aspects, including the temporal trend, of hospital morbidity from oral and oropharyngeal cancer in Brazil, considering hospitalizations in a health network associated with INCA.

## Method ethical aspects

This study was exempted from the analysis by the Ethics Committee because it used data obtained from public and unrestricted access databases. This study is reported in accordance with the STROBE (Strengthening the reporting of observational studies in epidemiology) guidelines [18].

### Study design and context

This is an observational study of temporal series, in which data on hospital admissions due to oral and oropharyngeal cancers were analyzed, obtained from the Information System of the Cancer Hospital Registry (SisRHC, Sistema de Informação do Registro Hospitalar do Câncer) in Brazil [17].

The study covers data on hospitalizations for oral cancer throughout Brazil between the years 2000 and 2015. The database was acquired through the IRHC/INCA website (download of databases containing occurrences in all states of the country, per year, from 2000 to 2015) and was organized and tabulated in MS Excel software.

All reported cases of hospitalization and classified during the first hospital visit as malignant neoplasms with primary location in the lips, oral cavity, salivary glands and oropharynx (ICD-10 codes C00-C10), according to the INCA [1] classification, were included in the study. Therefore, no sample calculation was performed.). In this study, hospital morbidity was related to the number of hospital admissions recorded in the Integrating Data System of the National Cancer Institute (INCA).

### Analyzed variables

Table 1 shows the variables analyzed in this study, their descriptions and the statistical treatment applied to each one.

**Table 1:** Variables, descriptions and statistical treatments shown in the database of the Information System of the Cancer Hospital Registry. Brazil, 2000–2015

Variables	Description and statistical treatment
In-hospital morbidity rate	Related to the number of hospital admissions for oral and oropharyngeal cancers per 100,000 inhabitants in the studied period, per macroregion of Brazil. The data related to the Brazilian population necessary for the calculation were obtained from the SUS IT Department (DATASUS), which aggregates information from the Demographic Censuses, Intercensus Projections and IBGE Population Estimates. The coefficients were later standardized by gender and age group (0 to 4; 5 to 9; 10 to 14; 15 to 19; 20 to 29; 30 to 39; 40 to 49; 50 to 59; 60 to 69; 70 to 79 years and 80 years and over), by the direct method, using as a standard the distribution of the world’s population created by the World Health Organization for this purpose [19]. The standardization aims to consider and remove the effect of factors related to the population distribution that interfere with the risk of developing the disease, allowing comparisons to be made
Anatomical site (originally described in IRH/INCA as “LOCTUDET”)	The codes were grouped by anatomical region for better data presentation. Thus, for this study, the lesions were presented as: “lip” – originally classified at the IRH/INCA as C00 (lip); “tongue” – originally C01 (base of the tongue) and C02 (other parts and unspecified parts of the tongue); “other parts of the mouth” – originally C03 (gingiva), C04 (floor of the mouth), C05 (palate) and C06 (other unspecified parts of the mouth: mucosa, vestibules, retromolar area); “salivary glands” – originally C07 (parotid gland) and C08 (other major salivary glands and unspecified major salivary glands); and "oropharynx" -originally C09 (palatine tonsil) and C10 (oropharynx) [13, 20]
Macroregion	Variable organized according to the distribution of the Brazilian Institute of Geography and Statistics (IBGE, Instituto Brasileiro de Geografia e Estatística) as North, Northeast, Southeast, South and Midwest regions [21]. This variable was identified based on the hospitalized patient's place of residence, as described in the notification form
Gender	Variable categorized into female, male and no information
Skin Color	Variable categorized as white, black, yellow, brown, indigenous or without information, according to the classification of the Brazilian Institute of Geography and Statistics (IBGE)
Level of schooling	Variable categorized according to the presentation in the original database as none, incomplete elementary level, complete elementary level, high school level, higher education level or no information
Age range	Variable categorized as 20–29, 30–39, 40–49, 50–59, 60–69, 70–79 years old, 80 years old and over and “no information”, considering the sum of these age groups as the total number of cases. This classification was chosen for age group presentation, as the assessed pathology shows a relevant incidence from adulthood onwards. There was a disproportionate frequency of cases classified as 0 years old, which were considered “no information”, indicating problems with registration in the database
Alcohol consumption	Variable categorized into: never consumed, ex-consumer, consumer and not evaluated
Smoking status	Variable recategorized according to the original database into: never smoked, ex-smoker, smoker and not evaluated (the latter also included—not applicable and no information)
Macroregion	Variable organized according to the distribution of the Brazilian Institute of Geography and Statistics (IBGE, Instituto Brasileiro de Geografia e Estatística) as North, Northeast, Southeast, South and Midwest regions [21]. This variable was identified based on the hospitalized patient's place of residence, as described in the notification form
Gender	Variable categorized into female, male and no information
Skin Color	Variable categorized as white, black, yellow, brown, indigenous or without information, according to the classification of the Brazilian Institute of Geography and Statistics (IBGE)
Level of schooling	Variable categorized according to the presentation in the original database as none, incomplete elementary level, complete elementary level, high school level, higher education level or no information
Age range	Variable categorized as 20–29, 30–39, 40–49, 50–59, 60–69, 70–79 years old, 80 years old and over and “no information”, considering the sum of these age groups as the total number of cases. This classification was chosen for age group presentation, as the assessed pathology shows a relevant incidence from adulthood onwards. There was a disproportionate frequency of cases classified as 0 years old, which were considered “no information”, indicating problems with registration in the database
Alcohol consumption	Variable categorized into: never consumed, ex-consumer, consumer and not evaluated
Smoking status	Variable recategorized according to the original database into: never smoked, ex-smoker, smoker and not evaluated (the latter also included—not applicable and no information)

### Statistical analysis

With the exception of the in-hospital morbidity rates, data were presented as relative frequency (percentage of cases). To analyze the trend of the in-hospital morbidity rates, generalized linear regression was used with the Prais-Winsten method, which allows first-order autocorrelation correction to be carried out in the analysis of series of values organized in time. This procedure allowed classifying the rates as increasing (*p* < 0.05 and positive regression coefficient), decreasing (*p* < 0.05 and negative regression coefficient) or stationary (*p* > 0.05) and enabled the quantification of annual averages of increase or decrease of the coefficients (annual percent change -APC) and its 95% confidence interval (95% CI) [22].

This technique was applied on the logarithm of rates. In the analysis of trend and annual variation, the coefficients were unstable in the first and last two years of the “2000 to 2015” series. Thus, it was decided to conduct the temporal trend analysis, excluding the years 2000, 2001, 2014 and 2015. Statistical analyzes were performed using the Stata^®^ software, version 14.0, and the graphs were prepared using the R^®^ software, version 3.5.0.

## Results

There were 121,971 hospitalizations for oral and oropharyngeal cancers from 2000 to 2015 in Brazil. Of these cases, 76.40% were male, 31.09% were aged between 50 and 59 years, 32.23% were white and 36.58% had incomplete elementary education. Moreover, the neoplasms classified as occurring in other parts of the mouth at the time of the first consultation were the most frequent cause of hospitalization (32.68%) and tumors already diagnosed with metastasis were more frequent (13.61%) than tumors with less severe staging (0.52%).

Table 2 shows the distribution of hospital admissions by case (C00 to C10) according to the macroregion of the notifying city and by gender, anatomical site, skin color, level of schooling, age group, alcohol and tobacco consumption.

**Table 2:** Distribution of hospital admissions due to oral and oropharyngeal cancers; percentage by anatomical site, gender, skin color, level of schooling, age group, alcohol and tobacco consumption, by macroregion. Brazil, 2000–2015

Variables	Macroregion (%)					Total Brazil	
	North	Northeast	Southeast	South	Midwest	n	%
*Anatomical site*							
Lip	3.83	6.29	6.81	8.32	3.95	8315	6.82
Tongue	27.04	28.49	29.44	27.73	31.24	35,240	28.89
Other parts of mouth	38.14	35.89	31.75	30.49	33.87	39,855	32.68
Salivary glands	10.60	8.93	6.69	8.49	8.61	9365	7.68
Oropharynx	20.39	20.40	25.30	24.97	22.32	29,196	23.94
*Gender*							
Male	67.33	68.65	78.69	80.30	77.61	93,184	76.40
Female	32.67	31.34	21.30	19.69	22.39	28,780	23.60
No information	0.00	0.01	0.00	0.01	0.00	7	0.01
*Skin color*							
White	21.81	17.34	21.44	83.56	36.41	39,313	32.23
Black	4.97	4.75	5.21	3.62	6.05	5894	4.83
Yellow	0.70	1.09	0.23	0.40	0.91	590	0.48
Brown	55.21	67.54	16.82	4.65	41.74	33,677	27.61
Indigenous	0.29	0.17	0.05	0.03	0.17	99	0.08
No information	17.02	9.10	56.24	7.74	14.72	42,393	34.76
*Level of schooling*							
None	18.93	23.49	23.49	5.90	12.19	14,705	12.06
Incomplete Elementary	40.05	30.90	30.90	36.36	25.46	44,622	36.58
Complete Elementary	11.03	9.00	9.00	17.47	13.20	17,006	13.94
High School	9.46	6.76	6.76	9.26	6.21	10,435	8.56
Higher Education	2.46	1.70	1.70	2.74	1.82	3436	2.82
No information	18.03	28.12	28.12	28.26	41.10	31,767	26.04
*Age range*							
20–29 years	2.29	1.37	0.99	1.25	1.50	1423	1.17
30–39 years	5.05	4.06	3.67	3.41	5.14	4586	3.78
40–49 years	15.68	14.49	17.86	17.61	19.97	20,663	17.05
50–59 years	26.01	25.95	32.57	33.69	31.52	37,678	31.09
60–69 years	23.25	24.74	25.06	25.56	23.54	30,287	24.99
70–79 years	17.62	18.42	13.93	13.48	13.59	18,112	14.94
80 years or older	9.95	10.97	5.92	5.00	4.74	8435	6.96
No information	0.15	0.01	0.00	0.00	0.00	10	0.01
*Alcohol consumption*							
Never	25.65	23.78	10.25	18.08	13.81	18,560	15.22
Ex-consumer	9.32	6.75	5.32	8.73	6.01	7795	6.39
Yes	37.41	28.07	22.08	29.46	25.43	30,828	25.27
Not assessed	27.62	41.41	62.3	43.73	54.75	64,788	53.12
*Smoking*							
Never	16.5	15.7	7.05	11.15	10.13	12,278	10.07
Ex-smoker	12.66	8.38	4.6	7.5	5.54	7586	6.22
Yes	52.4	39.41	27.42	43.12	34.79	41,281	33.84
Not assessed	18.44	36.51	60.93	38.24	49.54	60,826	49.87

The highest mean in-hospital morbidity rate for men was identified in the Southeast region (9.36/100,000 inhab.), while for women, it was identified in the Northeast region (2.29/100,000 inhab.). The trend of in-hospital morbidity rates was increasing in Brazil, for the entire population and by gender, as well as in the Northeast, South and Midwest regions.

Table 3 shows the standardized in-hospital morbidity rates due to oral and oropharyngeal cancers created to present an equitable distribution of hospital admissions for cases notified to INCA between the regions, considering the differences in population distribution.

**Table 3:** Adjusted in-hospital morbidity rates due to oral and oropharyngeal cancers per 100,000 inhabitants, by macroregion, by gender. Brazil, 2000–2015

Year	Macroregion						Brazil					
	North		Northeast		Southeast		South		Midwest		Total	
	Fem	Male	Fem	Male	Fem	Male	Fem	Male	Fem	Male	Fem	Male
2000	0.25	0.89	1.14	2.13	1.96	8.38	0.34	1.81	0.43	1.38	1.32	4.83
2001	1.85	2.53	1.91	4.04	2.12	9.35	0.62	2.67	0.36	1.14	1.71	5.99
2002	1.47	3.64	1.72	4.03	2.01	9.48	1.31	5.27	0.57	1.38	1.71	6.55
2003	1.83	3.42	1.91	4.58	2.11	9.62	1.05	4.96	0.65	2.1	1.79	6.74
2004	1.74	3.79	2.24	5.33	2.32	9.95	1.51	6.77	0.57	2.19	2.05	7.40
2005	2.01	3.08	2.19	5.93	2.44	10.22	1.92	9.40	1.09	2.61	2.20	8.09
2006	1.71	3.78	2.60	6.35	2.12	9.75	2.03	10.60	0.82	3.02	2.14	8.23
2007	1.28	3.46	2.24	5.90	2.15	9.42	1.85	10.21	1.06	4.42	2.01	8.01
2008	1.98	3.43	2.70	6.99	2.11	9.34	2.08	10.37	0.86	3.22	2.17	8.18
2009	1.19	3.47	2.84	7.27	2.18	9.42	2.31	10.51	1.13	3.45	2.25	8.33
2010	1.74	3.00	2.66	7.36	2.25	10.03	2.66	12.35	1.13	4.00	2.32	8.88
2011	1.77	3.36	3.01	8.03	2.49	10.77	2.62	11.56	0.90	3.29	2.51	9.22
2012	1.92	4.31	2.74	7.56	2.23	9.94	2.56	11.08	0.85	3.69	2.31	8.73
2013	1.50	3.14	2.80	8.24	2.31	9.65	2.45	10.89	0.84	2.90	2.32	8.64
2014	1.32	2.54	2.06	5.96	1.86	7.77	2.42	10.63	0.68	2.85	1.89	7.13
2015	1.36	2.59	1.89	6.14	1.53	6.70	2.05	9.36	0.30	1.61	1.61	6.37
Mean	1.56	3.15	2.29	5.99	2.14	9.36	1.86	8.65	0.77	2.70	2.02	7.58

Fem, female gender; Male, male gender

Table 4 shows the trend of in-hospital morbidity rates of oral and oropharyngeal cancers, between 2002 and 2013, by macroregion and the total of Brazil, by gender and by overall distribution. The temporal trend of the adjusted in-hospital morbidity rates was increasing in the Northeast, South and Midwest regions for males, whereas, for females, it was increasing in the Northeast and South regions.

**Table 4:** Trend and annual variation (APC—annual percent change) of the adjusted in-hospital morbidity rates due to oral and oropharyngeal cancers, by macroregion, gender and by overall distribution. Brazil, 2002–2013*

Variables	APC (%)	CI (95%)		*P*-value	Trend
		Lower	Upper		
*Both genders*					
North	− 0.18	− 1.42	1.07	0.75	Stable
Northeast	5.67	3.90	7.47	< 0.001	Increasing
Southeast	0.38	− 0.64	1.41	0.43	Stable
South	6.97	1.83	12.37	0.01	Increasing
Midwest	5.97	0.40	11.86	0.04	Increasing
Total Brazil	2.62	1.20	4.06	0.00	Increasing
*Male gender*					
North	− 0.08	− 1.38	1.23	0.89	Stable
Northeast	6.22	4.36	8.12	< 0.001	Increasing
Southeast	0.26	− 0.74	1.27	0.57	Stable
South	7.03	1.50	12.86	0.02	Increasing
Midwest	6.70	0.73	13.03	0.03	Increasing
Total Brazil	2.60	1.14	4.07	0.00	Increasing
*Female gender*					
North	− 0.30	− 2.55	2.00	0.77	Stable
Northeast	4.34	2.74	5.96	< 0.001	Increasing
Southeast	0.81	− 0.44	2.07	0.18	Stable
South	7.26	3.90	10.74	0.00	Increasing
Midwest	3.85	− 0.59	8.48	0.08	Stable
Total Brazil	2.68	1.26	4.11	0.00	Increasing

*As the rates were unstable in the first and last years of the “2000 to 2015” series, it was decided to carry out the trend analysis without the years 2000, 2001, 2014 and 2015 [18]

**Fig. 1:**
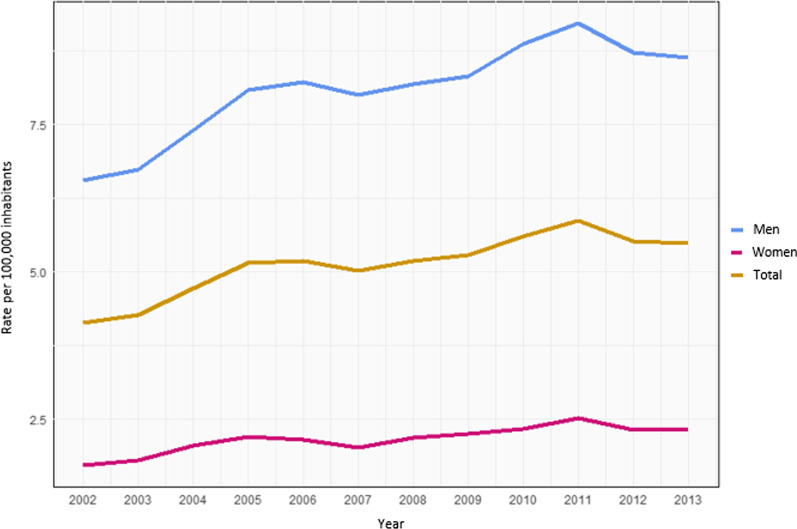
Trend of adjusted in-hospital morbidity rates of oral and oropharyngeal cancers, per 100,000 inhabitants, by gender. Brazil, 2002–2013*. *Note: As the coefficients were unstable in the first and last years of the “2000 to 2015” series, it was decided to carry out the trend analysis without the years 2000, 2001, 2014 and 2015 [22]

Figure 1 depicts the 2002-2013 temporal series, showing the trend of the adjusted in-hospital morbidity rates of oral and oropharyngeal cancers in Brazil (total), by gender.

## Discussion

This study identified that cases of hospital admissions due to oral and oropharyngeal cancers in Brazil, from 2000 to 2015 were mostly men. There is a predominance of individuals aged between 50 and 59 years, with low education and white or brown skin color. A greater number of cases with more advanced stage tumors classified as metastasis were observed. In addition, the temporal trend of in-hospital morbidity rates for oral and oropharyngeal cancer in the period 2002-2013 was increasing, both for males and females. The analysis of this information is unprecedented, considering the hospital morbidity of this disease in Brazil.

The factors smoking and alcohol consumption were relevant for the development of the disease among hospitalized patients in all regions. This fact can be evidenced by the greater number of cases with consumption or ex-consumption of alcohol than those who reported never having consumed alcohol or tobacco products. In addition, greater consumption of alcohol-tobacco [23, 24] has been associated with a higher prevalence of the disease, especially in female patients.

Cases diagnosed in advanced staging (with metastasis) at the time of first admission had higher percentages than cases classified as early staging with localized involvement. Data from the INCA Hospital Cancer Records warn that, as most patients arrive at hospitals at an advanced stage of the disease, the treatment is no longer curative, being in most cases mutilating [25].

These data are relevant, as the diagnosis in early stages is the strongest predictor of survival for head and neck squamous cell carcinoma (among which oral in oropharyngeal cancer stands out) in South American countries such as Brazil, Argentina, Uruguay and Colombia [26], which may interfere with the mortality rates of this disease. From this perspective, the progression of the disease staging may imply extensive or intensified treatments that cause the loss of functionally important tissues, implying significant morbidity and worse functional and psychosocial oncological results [27, 28].

It was found that the trend in hospital morbidity rates for oral and oropharyngeal cancer was increasing for both males and females, as well as for both genders together in the analyzed period of time. Following this finding, another study also found a growing trend when it analyzed the granting of social security benefits in Brazil for oral cavity cancer (2006–2013) [29].

The growing trend in the evidenced overall picture of hospitalizations may be linked to the initial hypothesis of better case registration after the consolidation of the nationwide hospital integrated information system. As the analysis of these data is unprecedented in the literature, it was not possible to compare them with other studies on hospitalization trends due to oral and oropharyngeal cancers. However, considering the results of studies that indicate a stability or decrease in mortality rates related to neoplastic conditions of the oral cavity [31, 32] in contrast to the growing trend of hospitalization observed in the present study, this research suggests better access to health services over time series.

In this way, users of the public health system who previously did not have access to treatment can have access to the hospital network and try to be cured [24]. This reasoning would also be compatible with the results that the majority of neoplasms present with advanced staging. As it is a chronic disease, people would reach the health service network with a previously established severity of the case [30].

It is worth mentioning that the inclusion and expansion of the family health network in Brazilian communities has shown the potential to reach the population at risk for the development of oral and oropharyngeal cancer [31, 32]. In this sense, a recent study shows that the inclusion of oral health teams is associated with early and timely detection of these diseases [12].

This expansion of coverage of primary care combined with a greater possibility of access to treatments in the hospital network, would result in lower mortality rates but in greater in-hospital morbidity [33]. Another study indicated the association between the increase in the number of oral health teams and the timely diagnosis of oral and oropharyngeal cancers [12].

Therefore, it is reiterated that studies of epidemiological surveys for oral cancer diagnosis and surveillance are of utmost importance, allowing better management of public policies in the health area and data that support planning by health professionals, so that users have greater chances of treatment and cure [34, 35].

As Brazil is a country with a large territorial extension and has many regional variations, both geographical and populational, the importance of developing research by state to assess the specific characteristics of its population is highlighted. Thus, this study met its purpose of identifying trends and characterizing epidemiological aspects related to morbidity of oral and oropharyngeal cancers in Brazil, showing data that had not been published before in the literature.

We point out some limitations of the present study, since by using public information systems, it was observed that some data were under-recorded (classified as not applicable or missing), which seems to correspond to the failure to complete the medical records at the time of hospitalization and also the migration of some databases from regional platforms to a national platform, particularly in the state of São Paulo, which has a single state database [19].

Despite this, the data presented here are considered to be the best and most comprehensive information on morbidity from oral and oropharyngeal cancer available in Brazil. In addition, these data are already consolidated and have a policy for monitoring and checking information at the state and national levels to avoid duplication of information [17], providing them with reliability and robustness.

Our results demonstrate that greater attention should be paid to the early detection and treatment of oral and oropharyngeal cancers, reinforcing that dedicated professional action is still required in the fight against the disease and educating the population about risk factors and signs and symptoms of oral and oropharyngeal cancers. Also, it is necessary to expand cancer care, by involving the Primary Health Care level and high complexity centers.

We conclude that the distribution of in-hospital morbidity rates for oral and oropharyngeal cancers in the country is irregular. There is a predominance of male hospitalized cases and predominantly located in more developed places, such as in the Southeast and South regions. In addition, there was a greater trend in the hospital morbidity rate for these neoplasms in both genders and for the entire country.

## Data Availability

The datasets generated and analyzed during the current study are available in the Hospital Cancer Registry of the National Cancer Institute of Brazil (RHC-INCA, in Portuguese abbreviation) repository, available in https://irhc.inca.gov.br/RHCNet/visualizaTabNetExterno.action.
